# WGA-based lectin affinity gel electrophoresis: A novel method for the detection of O-GlcNAc-modified proteins

**DOI:** 10.1371/journal.pone.0180714

**Published:** 2017-07-07

**Authors:** Yuji Kubota, Ko Fujioka, Mutsuhiro Takekawa

**Affiliations:** 1 Division of Cell Signaling and Molecular Medicine, Institute of Medical Science, The University of Tokyo, Tokyo, Japan; 2 Department of Computational Biology and Medical Sciences, Graduate School of Frontier Sciences, The University of Tokyo, Tokyo, Japan; Center for Cancer Research, UNITED STATES

## Abstract

Post-translational modification with O-linked β-N-acetylglucosamine (O-GlcNAc) occurs selectively on serine and/or threonine residues of cytoplasmic and nuclear proteins, and dynamically regulates their molecular functions. Since conventional strategies to evaluate the O-GlcNAcylation level of a specific protein require time-consuming steps, the development of a rapid and easy method for the detection and quantification of an O-GlcNAcylated protein has been a challenging issue. Here, we describe a novel method in which O-GlcNAcylated and non-O-GlcNAcylated forms of proteins are separated by lectin affinity gel electrophoresis using wheat germ agglutinin (WGA), which primarily binds to N*-*acetylglucosamine residues. Electrophoresis of cell lysates through a gel containing copolymerized WGA selectively induced retardation of the mobility of O-GlcNAcylated proteins, thereby allowing the simultaneous visualization of both the O-GlcNAcylated and the unmodified forms of proteins. This method is therefore useful for the quantitative detection of O-GlcNAcylated proteins.

## Introduction

Protein post-translational modifications (PTMs) regulate various properties of proteins such as stability, subcellular localization, and catalytic activity. Of these PTMs, O-linked β-N-acetylglucosamine modification (O-GlcNAcylation) is a type of protein glycosylation in which a single-sugar, N-acetylglucosamine, is added to the hydroxyl moiety of serine and threonine residues of cytoplasmic and nuclear proteins. Previous studies have shown that thousands of proteins in cells are modified with O-GlcNAc [1]. Protein O-GlcNAcylation is dynamically and reversibly regulated by the paired enzymes, O-GlcNAc transferase (OGT) and O-GlcNAcase (OGA) [2, 3]. OGT catalyzes the addition of a GlcNAc moiety from a uridine diphosphate (UDP)-GlcNAc to target proteins, while OGA removes the O-GlcNAc from the modified proteins. In contrast to protein phosphorylation that is regulated by many kinases and phosphatases, OGT and OGA are the only enzymes responsible for the protein O-GlcNAcylation cycle in a cell. Since both O-GlcNAcylation and phosphorylation occur at serine and threonine residues, these two types of PTMs are mutually exclusive on the same target residue. In addition, these modifications can suppress each other within a protein even when they do not take place on the same residues [4]. This dynamic crosstalk between phosphorylation and O-GlcNAcylation has been frequently found in various cellular proteins, and the molecular function of the modified proteins dramatically changes according to the modification state [4].

Recent increases in the sensitivity and rapidity of mass spectrometry have enabled high-throughput screening of a wide variety of PTMs including O-GlcNAc-modification [5]. However, stoichiometric analysis of O-GlcNAcylation has been reported for only a few proteins because the conventional methods for quantifying the O-GlcNAcylation level entail laborious processes and high-running cost [6, 7]. In addition, in collision-induced dissociation (CID) for mass-spectrum analysis, O-GlcNAc is readily released from the conjugated peptide [8], which limits evaluation of the O-GlcNAcylation level of a specific protein. The development of novel techniques for quantitative analysis of protein O-GlcNAcylation therefore remains an important challenge.

A previous study demonstrated that performance of SDS-PAGE with a specific lectin-gel layer consisting of a concanavalin A (ConA)-copolymerized acrylamide gel, allowed the separation of the N-glycosylated form of a transmembrane protein, anion exchanger 1 (AE1), from the unmodified form [9]. Since ConA exhibits high affinity for α-D-mannosyl and α-D-glucosyl residues in glycoproteins, the immobilized-ConA causes retardation of the glycosylated form(s) of the protein during electrophoresis, thereby allowing evaluation of the protein glycosylation level by quantification of the degree of mobility shift. Based on the principle of this system, we developed a novel method for the separation of cytoplasmic and nuclear O-GlcNAcylated proteins by using wheat germ agglutinin (WGA), a lectin from *Triticum vulgaris* [10]. The physiologically active form of WGA exists as a homodimer that has multiple GlcNAc-binding sites [11, 12]. Analysis of the crystal structure of WGA showed head-to-tail interaction between two WGA molecules. Homodimerized WGA is highly resistant to protein denaturing conditions such as high temperature, acidic environments, and chaotropic agents [13, 14]. Due to these physicochemical features, WGA has long been used as an investigative tool for O-GlcNAc proteins. For example, a horseradish peroxidase (HRP)-conjugated WGA can be utilized as a probe to detect O-GlcNAcylated proteins in western blotting, and a WGA-agarose column can be used for the purification and concentration of O-GlcNAcylated proteins from cell extracts. In addition, fluorescein isothiocyanate (FITC)-labeled WGA can be used to visualize a specific banding pattern of highly condensed chromatin at which GlcNAc-containing proteins are localized [15]. Here, we describe a novel electrophoretic method, termed WGA-SDS-PAGE, that separates O-GlcNAcylated and unmodified proteins, thereby enabling quantification of O-GlcNAcylated proteins.

## Materials and methods

All reagents and solvents in this study were analytical grade or better.

### Plasmids

The expression plasmids for Tab1, TFG, UBAP2L, TSC22D1, OGA and OGT were generated using pcDNA3HA, pcDNA4Myc and pcDNA3Flag vectors. All expression plasmids were purified using a commercially available plasmid purification kit (Roche, Plasmid Midi-prep).

### Media and buffers

DMEM (Nacalai Tesque, Japan) was supplemented with 10% fetal bovine serum (FBS), L-glutamine, streptomycin and penicillin. Stock lysis buffer contained 20 mM Tris-HCl (pH 7.5), 137 mM NaCl, 1% Triton X-100, and 10% glycerol. The following chemicals were added to the stock lysis buffer immediately before harvesting the cells: 1 mM dithiothreitol; 0.5% deoxycholate (DOC); 50 μM PUGNAc (Sigma), 10 μM Thiamet-G (TMG); 1 mM phenylmethylsulphonyl fluoride (PMSF); 10 μg/ml aprotinin; and 10 μg/ml leupeptin. Stock lysis buffer containing 0.5% DOC and 0.1% SDS was used for washing immunoprecipitates. SDS-PAGE loading buffer contained 65 mM Tris-HCl (pH 6.8), 5% 2-mercaptoethanol, 10% glycerol, 3% SDS, and 25 μg/ml bromophenol blue. Stock separating gel buffer contained 1.5 M Tris-HCl (pH 8.8) and 0.4% SDS, and stock stacking gel buffer contained 0.5 M Tris-HCl (pH 6.8) and 0.4% SDS. WGA protein (J-OIL MILLs, lot numbers: 91014C and 11213C) was dissolved in 10 mM Tris-HCl (pH 7.5) to give a final concentration of 10 mg/ml. Transfer buffer for electroblotting of O-GlcNAcylated proteins contained 25 mM Tris, 192 mM glycine, 20% methanol, and 0.1% SDS.

### Antibodies

The following antibodies were used: anti-HA monoclonal antibody (mAb) F-7 (Santa Cruz, sc-7392); anti-Myc mAb 9E10 (Santa Cruz, sc-40); anti-Myc polyclonal antibody (pAb) A14 (Santa Cruz, sc-789); mouse mAb M2 specific to the Flag epitope (Sigma, F1804); anti-Actin mAb (Wako, Japan, 013–24553); anti-AGFG1 pAb (Abcam, ab86349); anti-O-GlcNAc mAb (Santa Cruz, RL2: sc-59624, CTD110.6: sc-59623); anti-Nup62 pAb (Santa Cruz, sc-25523); and anti-OGT pAb (Abcam, ab96718). All antibodies were used at a dilution of 1:2,000 for immunoblotting. HRP-conjugated secondary antibodies specific for mouse or rabbit IgG were purchased from GE healthcare. WGA-HRP was purchased from J-OIL MILLS (Japan, J420). Succinylated WGA-HRP was purchased from EY-laboratories (H-2102-1, lot number: 330806–1).

### Cell culture, transfection and harvesting

HEK293 and COS-7 cells were purchased from RIKEN Cell Bank (Japan, HEK293: RCB1637, COS-7: RCB0539). For transient transfection of expression plasmids, 2x10^5^ HEK293 or COS-7 cells were seeded on 35 mm dishes and incubated overnight at 37°C with 5% CO2. The appropriate combinations of expression plasmids (total 1 μg/dish) were transfected into the cells using the X-tremeGENE 9 DNA transfection reagent (Sigma). The culture medium was changed 24-hours later. After an additional 24-hour incubation, the cells were washed twice with ice-cold PBS and harvested with Lysis buffer on ice. The cell lysates were centrifuged at 20,000 x *g* for 15 min at 4°C, and the supernatants were used for several assays.

### Knockdown of OGT with siRNA

One day before transfection, 5 x 10^4^ HEK293 cells were seeded on a 35-mm culture dish. A double-stranded small interfering RNA (siRNA) oligonucleotide specific for human OGT (Mission siRNA, SASI_Hs01_00141131; Sigma), was transfected into the cells with the Lipofectamine RNAiMAX Transfection Reagent (Thermo) according to the manufacturer’s protocol. Briefly, 4 μl of RNAiMAX was diluted in 500 μl of Opti-MEM I (Thermo), and was then mixed with the siRNA oligonucleotide suspended in 500 μl of Opti-MEM I. After incubation for 15 minutes at room temperature, the siRNA mixture was added to the cells. Seventy-two hours after transfection, the cells were harvested with Lysis buffer. MISSION siRNA Universal Negative Controls (Sigma) was used as a negative control for this experiment. All siRNA nucleotides were used at a final concentration of 50 nM. The efficacy of OGT expression knockdown was evaluated by immunoblotting with the specific antibody.

### Immunoprecipitation

Cell lysates were precleared by incubation with protein G-Sepharose beads at 4°C for 1 hr. The lysates were then incubated with an anti-HA or an anti-Myc Ab at 4°C for 3 hr with gentle rotation. Protein G-Sepharose beads were then added and incubated for 1 hour. The resulting immune-complexes were washed three times with stock lysis buffer containing 0.1% SDS. The immunoprecipitates were mixed with SDS loading buffer, and were completely denatured by boiling for 4 min. After centrifugation at 20,000 x g for 1 min, the supernatants were loaded onto the SDS-PAGE gel.

### Preparation of a WGA-polyacrylamide gel layer

A mini-type electrophoresis apparatus (gel thickness, 1 mm; Nihon-Eido, Japan) was used for these experiments. The separating polyacrylamide gel solution (7.5% acrylamide) was made by mixing 5 ml of 30% acrylamide solution (Nacalai Tesque, Japan), 5 ml of the stock separating gel buffer, 10 ml of deionized water, 90 μl of 10% ammonium persulfate solution (APS) and 30 μl of tetramethylethylenediamine (TEMED), and was poured between two glass plates. Following formation of the separating gel, the WGA-polyacrylamide gel solution was made by mixing 0.375 ml of a 10 mg/ml WGA solution, 0.25 ml of a 30% acrylamide solution, 0.25 ml of the stock separating gel buffer, 0.119 ml of deionized water, 4.5 μl of 10% APS and 1.5 μl of TEMED, and was poured on top of the separating gel. To make a horizontal gel surface, water-saturated isobutanol was layered onto the surface of the separating and WGA polyacrylamide gels. After gel polymerization, the stacking gel solution was made by mixing 1.5 ml of a 30% acrylamide solution, 2.5 ml of the stock stacking gel buffer, 6 ml of deionized water, 90 μl of 10% APS and 30 μl of TEMED, and was layered on top of the WGA-gel. A gel comb was then immediately inserted into the stacking gel solution. Gels constructed in the absence of the WGA layer are referred to as standard SDS-PAGE gels.

### Running of WGA-SDS-PAGE, and electroblotting onto a nitrocellulose membrane

Aliquots of whole cell lysates were denatured in SDS–PAGE loading buffer by boiling for 4 min, and were then separated by 7.5% SDS-PAGE according to Laemmli's procedure in the absence (standard SDS-PAGE) or presence of the WGA-gel. The electrophoresis was performed at 4°C at a constant current of 20 mA. After the electrophoresis was completed, the separating gel fused with the WGA-gel was washed three times with transfer buffer. Standard and WGA-SDS-PAGE gels were elecroblotted onto a nitrocellulose membrane in GlcNAc-free transfer buffer. Note that the addition of 0.5 M N-acetylglucosamine to the transfer buffer did not affect the transfer efficiency of O-GlcNAcylated proteins to a nitrocellulose membrane (S1 Fig).

### Immunoblotting analysis

For the detection of proteins, the nitrocellulose membranes were blocked with 2% ECL blocking agent (GE) for 1 hour at R.T. For the detection of O-GlcNAcylation, membranes were blocked with 3% BSA for 1 hour at R.T. After incubation of the membranes with the appropriate primary antibodies overnight at 4°C, antibody-bound proteins on the membranes were visualized by using enhanced chemiluminescence (ECL prime, GE). An aliquot of HEK293 cell lysates containing 30 μg protein was used for the detection of endogenous AGFG1 and Nup62. Digitized images were captured by LAS-1000 Plus with a CCD camera (Fujifilm), and protein bands were quantified by densitometric analysis using Image gauge (Fujifilm). The detection of O-GlcNAcylation using succinylated WGA-HRP was carried out as described previously [16].

## Results and discussion

### Affinity-based separation of O-GlcNAcylated proteins through an acrylamide gel containing polymerized WGA

**Fig 1:**
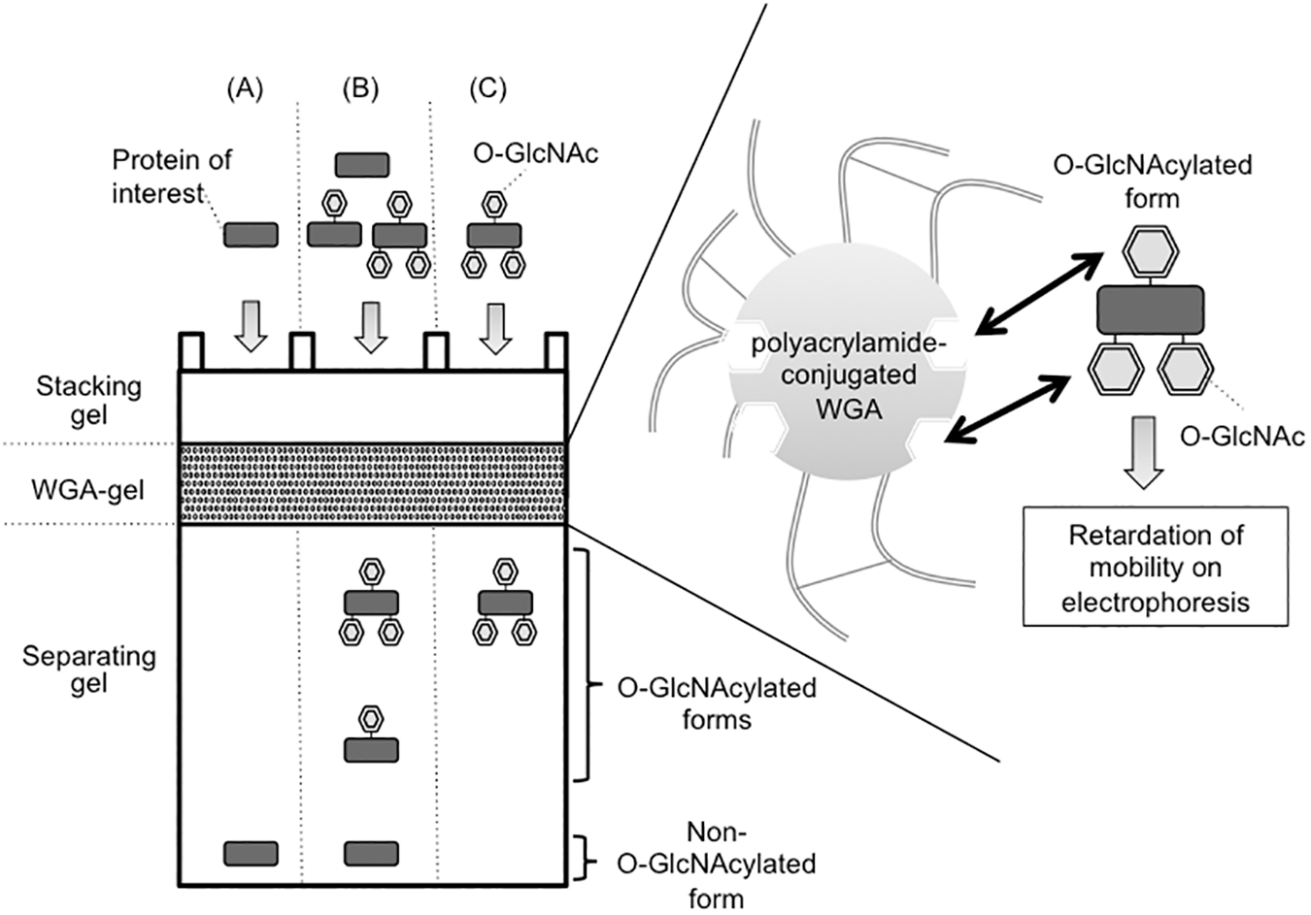
Schematic diagram of the principle of WGA-SDS-PAGE. A whole cell lysate contains proteins with different O-GlcNAcylation states ranging from no (A), through partial (B) to high (C) O-GlcNAcylation. In the WGA-copolymerized acrylamide gel layer, the migration of the O-GlcNAcylated proteins is retarded, thereby producing slower-migrating bands in the separating gel.

**Fig 2:**
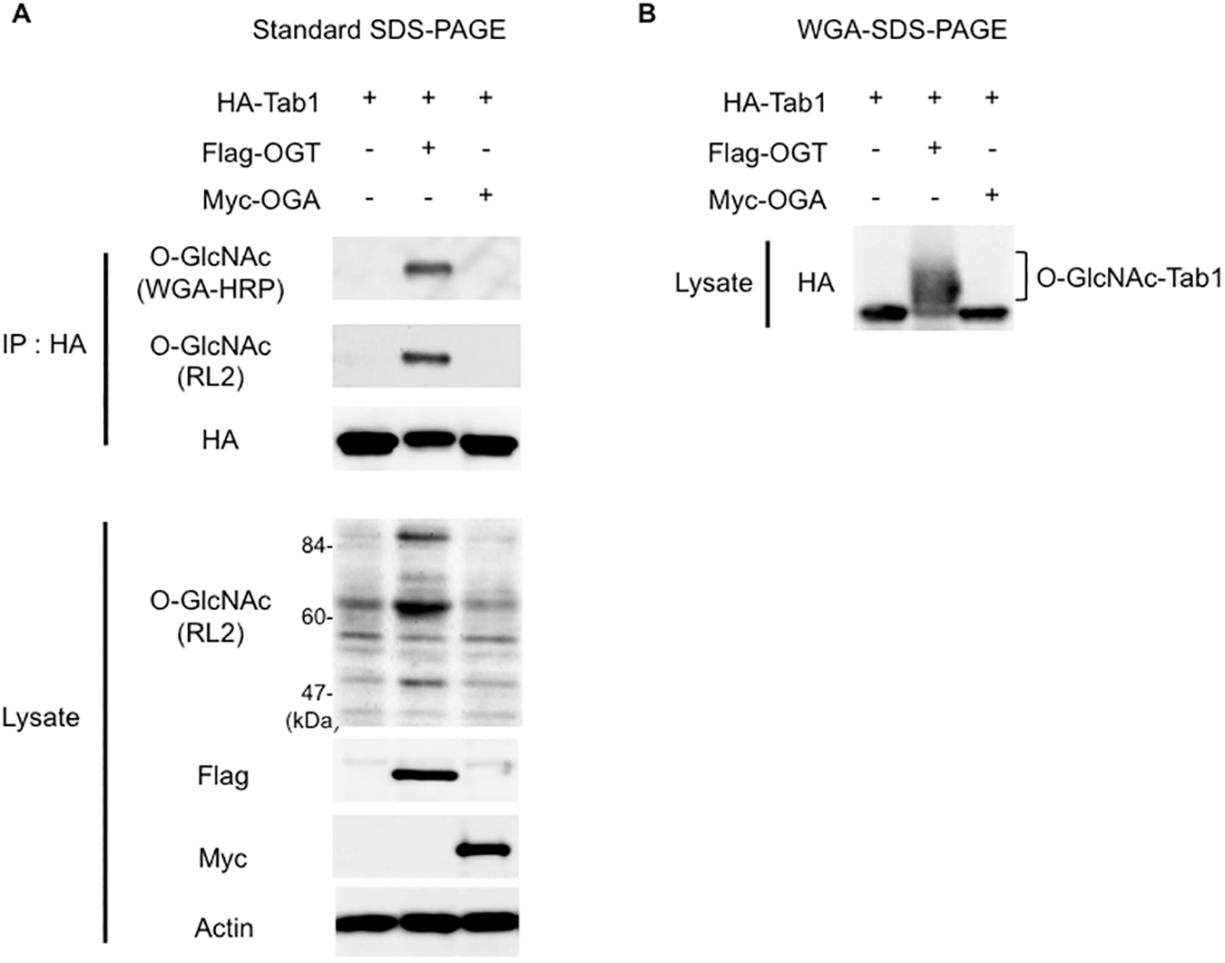
Separation and detection of O-GlcNAcylated HA-Tab1 by WGA-SDS-PAGE. (A) Ectopic expression of OGT augments O-GlcNAcylation of Tab1. HA-Tab1 was transiently expressed with either Flag-OGT or Myc-OGA in HEK293 cells. Immunoprecipitated (IP) HA-Tab1 was separated by standard [WGA(-)]-SDS-PAGE, and probed for O-GlcNAcylation using WGA-HRP (top panel) or the anti-O-GlcNAc RL2 Ab (2nd panel). The O-GlcNAcylation levels of proteins in the total cell lysates are also shown (4th panel). Actin was used as a loading control (bottom panel). (B) O-GlcNAcylated Tab1 forms are separated on WGA-SDS-PAGE. An aliquot of the whole cell lysates used in (A) was separated on WGA-SDS-PAGE, followed by immunoblotting with an anti-HA Ab (upper panel).

We prepared an acrylamide gel containing a layer of polymerized WGA between the stacking and the separating gels (Fig 1). As O-GlcNAcylated proteins pass through the WGA-containing layer of the gel, the O-GlcNAc residues interact with the immobilized-WGA, resulting in retardation of their mobility. To evaluate the efficacy of WGA-SDS-PAGE for the separation of O-GlcNAcylated proteins, we used Tab1, which is an activator of TAK1/MAP3K7 [17], as a representative O-GlcNAcylatable protein [18]. To enhance the O-GlcNAcylation level of Tab1, we expressed HA-tagged Tab1 together with human OGT in HEK293 cells. Immuno-purified HA-Tab1 was first separated in the absence of the WGA-gel layer by using standard SDS-PAGE, and the O-GlcNAcylation level of Tab1 was assessed by immunoblotting using either an anti-O-GlcNAc antibody (RL2) or WGA-HRP. As shown in Fig 2A, this analysis confirmed that coexpression of OGT significantly increased the O-GlcNAcylation level of Tab1. However, in HA-blotting following standard SDS-PAGE, the difference in the mobility of the O-GlcNAcylated Tab1 as compared with that of the non-O-GlcNAcylated Tab1 that was produced by its coexpression with OGA was barely discernable (Fig 2A, third panel). We next prepared a WGA-copolymerized acrylamide gel layer (WGA, 3.75 mg/ml; gel-length, 9 mm) between the stacking and the separating gels, and a small volume of the same whole cell lysates was subjected to WGA-SDS-PAGE. In this experiment, when HA-Tab1 was expressed alone or together with OGA, the resulting non-O-GlcNAcylated Tab1 appeared as a single band (Fig 2B). In contrast, when coexpressed with OGT, the O-GlcNAcylated Tab1 was separated into several bands with slower mobility during electrophoresis. Considering that proteins conjugated with multiple O-GlcNAc residues have high binding affinity for WGA [12], the relatively slower-migrating bands observed in WGA-SDS-PAGE are considered to be highly O-GlcNAcylated forms of Tab1. In addition, we observed that other O-GlcNAcylatable proteins (TFG, TSC22D1 and UBAP2L), which were previously identified through global O-GlcNAc proteome analyses [19–21], also showed slower-migrating bands in WGA-SDS-PAGE when co-expressed with OGT (S2 Fig). Since WGA binds not only N-acetylglucosamine but also sialic acid, we investigated if the slower-migrating proteins were indeed modified by O-GlcNAcylation. For this purpose, we used succinylated WGA-HRP (sWGA-HRP) to probe O-GlcNAcylated proteins since this chemically modified lectin selectively binds to GlcNAc but not to sialic acid [22]. As shown in the S2 Fig, the up-shifted bands of TFG, TSC22D1 and UBAP2L were detected by sWGA-HRP in WGA-SDS-PAGE. These data indicated that the slower-migrating bands of proteins in WGA-SDS-PAGE were derived from their O-GlcNAcylation.

**Fig 3:**
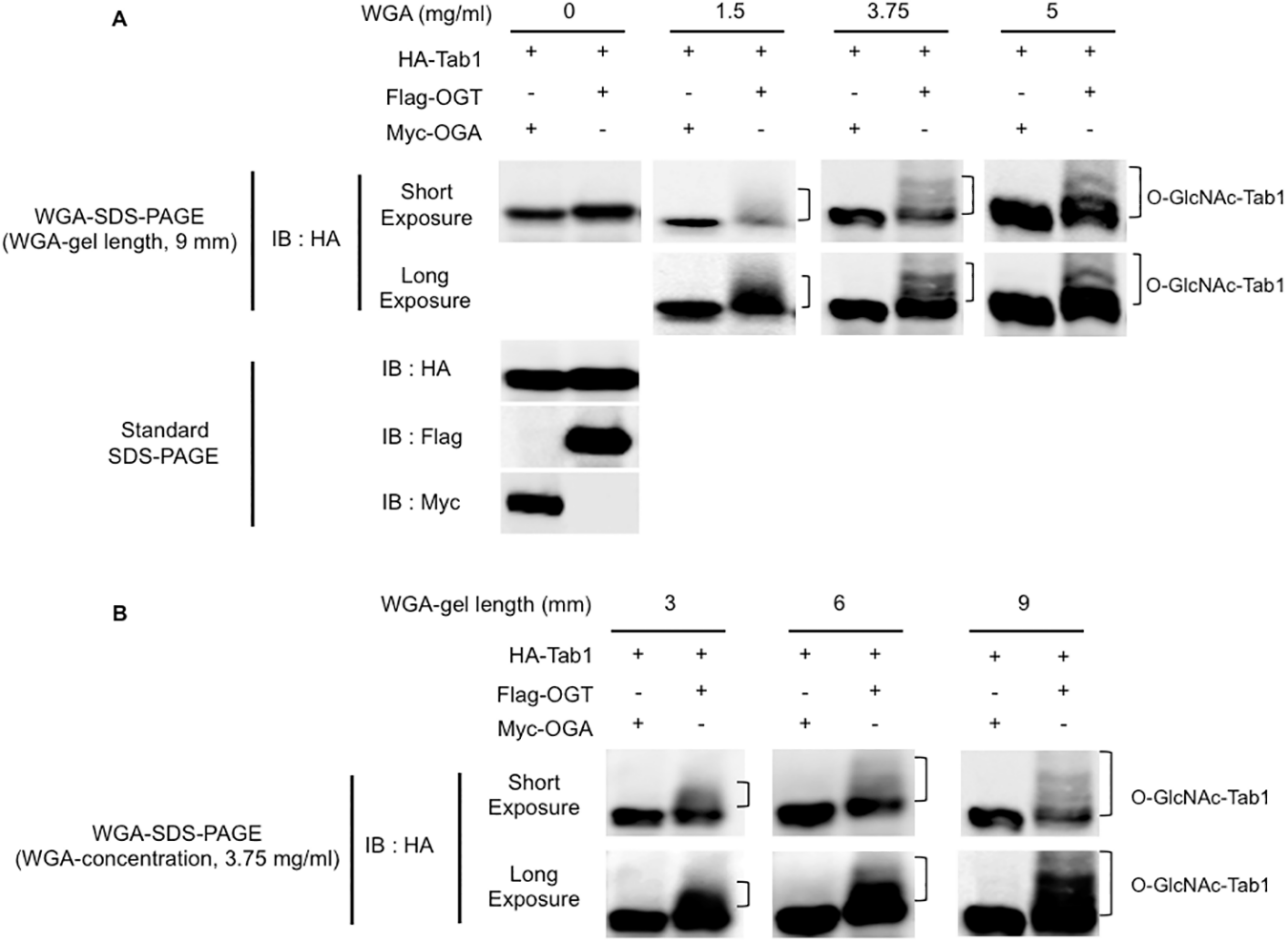
The effects of WGA concentration and length of the WGA-gel on the electrophoretic-mobility of O-GlcNAcylated proteins during WGA-SDS-PAGE. (A) An increase in WGA concentration improves the efficacy of separation of O-GlcNAcylated HA-Tab1. HA-Tab1 was co-expressed together with Flag-OGT or Myc-OGA in COS-7 cells. The cell lysates were separated by WGA-SDS-PAGE with gels containing the indicated concentrations of WGA, followed by immunoblotting with an anti-HA antibody (top and 2nd panels). The length of the WGA-gel layer was fixed at 9-mm. An aliquot of the same cell lysates was also separated with standard SDS-PAGE, and immunoblotted with an anti-HA antibody (3rd panel). The expression levels of Flag-OGT and Myc-OGA are also shown (4th and bottom panels). (B) Increase in the length of the WGA-gel layer enhances the efficacy of separation of O-GlcNAcylated Tab1 and the resolution of the protein bands. WGA-gel layers of different lengths (3, 6, and 9 mm) were prepared, and aliquots from the same cell lysate used in (A) were then separated on these WGA-SDS-PAGE gels. HA-Tab1 was detected with an anti-HA antibody as in (A). The WGA concentration was fixed at 3.75 mg/ml.

Next, to assess the effect of the WGA concentration in the gel on the mobility-shift of O-GlcNAcylated proteins, we prepared a series of WGA-gels containing various concentrations (0, 1.5, 3.75, or 5 mg/ml) of WGA, with a fixed WGA-gel length (9 mm). HA-Tab1 was exogenously expressed with OGT or OGA in COS-7 cells, and the whole cell lysates were run on the WGA-SDS-PAGE gels containing the different concentrations of WGA. As shown in Fig 3A, higher concentrations of WGA exaggerated the upward band-shift of O-GlcNAcylated Tab1. We next examined if the length of the WGA-gel affects the electrophoretic-mobility of O-GlcNAcylated proteins during WGA-SDS-PAGE. The cell lysates were run on WGA-SDS-PAGE with a WGA-gel layer of different lengths (3, 6, and 9 mm), but with the WGA concentration fixed at 3.75 mg/ml. Immunoblotting with an anti-HA antibody showed that the longest length (9 mm) of WGA-gel layer led to sharp and multiple HA-Tab1 bands with slower electrophoretic mobility (Fig 3B), while the shorter WGA-gel layer lengths caused diffusion of individual Tab1 bands. These data suggested that the efficacy of WGA-based electrophoretic separation of O-GlcNAcylated proteins depends on the concentration and the length of the WGA-gel. The best experimental conditions for the separation of the O-GlcNAcylated Tab1 proteins tested in this study were a 9 mm-gel length and more than 3.75 mg/ml of WGA, although these values may vary for individual O-GlcNAcylated proteins.

### The detection and separation of endogenously O-GlcNAcylated proteins by WGA-SDS-PAGE

**Fig 4:**
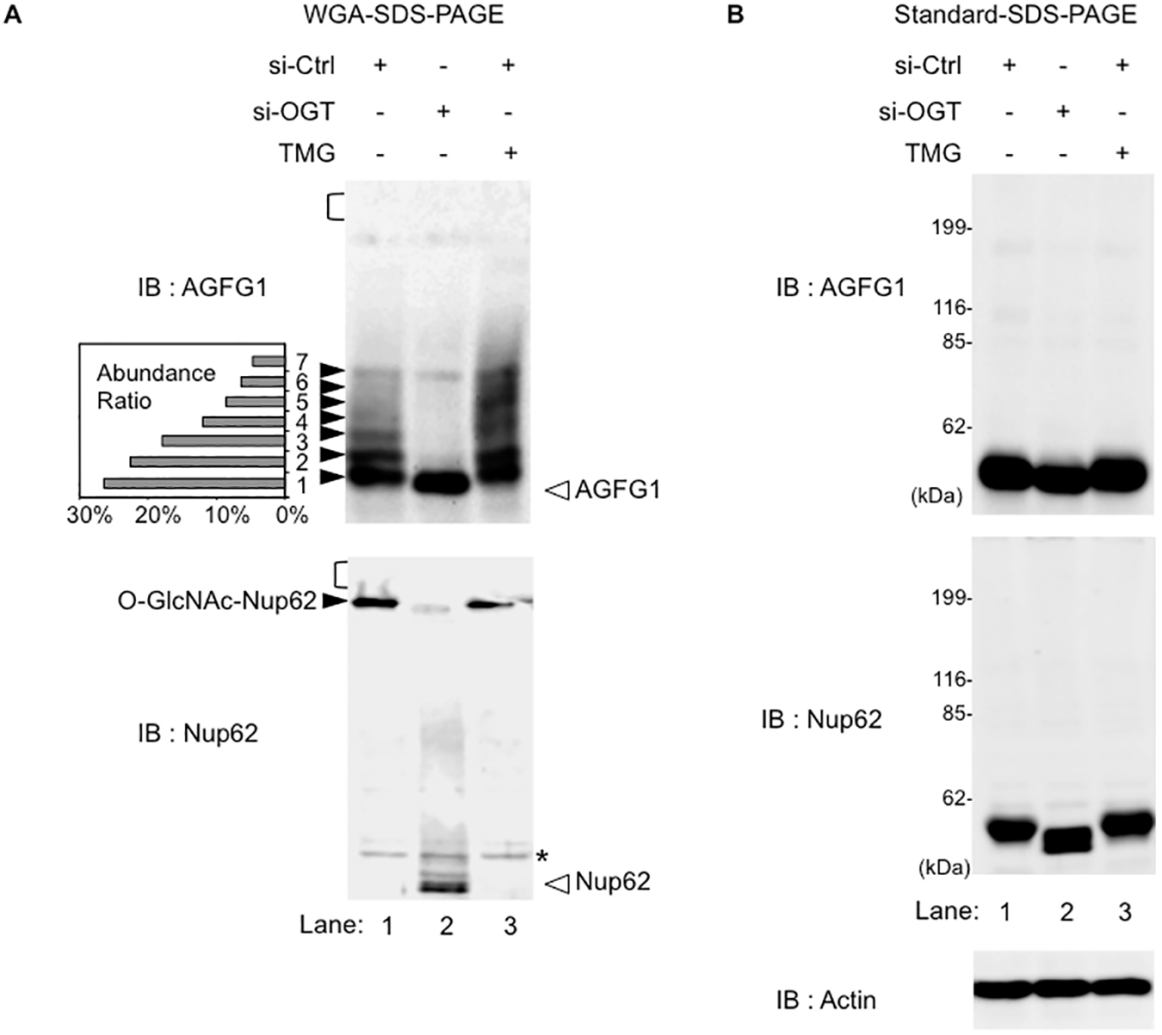
Detection and separation of endogenous O-GlcNAcylated proteins by WGA-SDS-PAGE. (A and B) Whole cell lysates from HEK293 cells transfected with siRNA targeting OGT or control siRNA, and treated with or without 10 μM Thiamet-G (TMG) for 24 hours were separated on WGA-SDS-PAGE (A) or standard SDS-PAGE (B). WGA-gel layer conditions were fixed at 9-mm in length and a concentration of 3.75 mg/ml. Immunoblotting was performed with an anti-AGFG1 antibody (upper panels) or an anti-Nup62 antibody (bottom panels). The black and white arrowheads indicate the O-GlcNAcylated and non-O-GlcNAcylated protein bands, respectively. Individual migration bands of AGFG1 (A, upper panel, no. 1–7) were quantified by densitometry, and the relative abundance ratio is shown in a small graph. The area of the WGA-gel layer transferred to the nitrocellulose membrane is indicated by a black bracket. Actin was used as a loading control (bottom panel, B).

We next examined if WGA-SDS-PAGE could be applied to the detection of endogenously O-GlcNAcylated proteins. For this purpose, we focused on the protein, ArfGAP with FG Repeats 1 (AGFG1), that plays a critical role in the traffic of human immunodeficiency virus (HIV)-derived RNA molecules from the nucleus to the cytoplasm [23–25]. Although previous studies have shown that AGFG1 can be O-GlcNAcylated in cells [26, 27], the ratio of O-GlcNAcylated AGFG1 to total AGFG1 under physiological conditions remains unknown. For this assay, an aliquot of the whole cell lysate of human HEK293 cells was separated using WGA-SDS-PAGE or conventional SDS-PAGE. As shown in Fig 4A (upper panel, lane 1), after separation on WGA-SDS-PAGE, endogenous AGFG1 exhibited multiple slower-migrating bands upon immunoblotting using an anti-AGFG1 antibody. In contrast, these bands were not observed when AGFG1 proteins were separated by SDS-PAGE in the absence of a WGA-gel layer (Fig 4B, upper panel, lane 1). To confirm that these slower-migrating bands were indeed O-GlcNAcylated forms of AGFG1, we blocked O-GlcNAcylation of AGFG1 by knockdown of OGT expression with siRNA. This siRNA induced a decrease in OGT expression in the cells, and, concomitant with this decrease, the O-GlcNAcylation levels of total cellular proteins significantly declined (S3 Fig, lane 2). Consistent with this result, the slower-migrating AGFG1 bands that were observed in WGA-SDS-PAGE almost completely disappeared when OGT was depleted with siRNA, and, instead, a single faster-migrating band was observed (Fig 4A, lane 2). Furthermore, treatment of HEK293 cells with a potent OGA inhibitor Thiamet-G (TMG), which increases O-GlcNAcylation levels of cellular proteins (S3 Fig, lane 3), enhanced the intensity of the upper-shifted AGFG1 bands in WGA-SDS-PAGE (Fig 4A, upper panel, lane 3). Densitometric analysis of the multiple AGFG1 bands demonstrated that only 26% of AGFG1 remained in the non-O-GlcNAcylated state in human HEK293 cells (Fig 4A, upper panel, lane 1), suggesting that most cellular AGFG1 proteins were modified with O-GlcNAc at multiple sites.

We also assessed the endogenous O-GlcNAcylation level of another representative O-GlcNAcylatable protein, Nup62 [28]. This protein is a component of the nuclear pore complex (NPC), and the bulk of its O-GlcNAcylated residues is in the FG-repeat domain [29, 30]. Western blot analysis of cell lysates separated on WGA-SDS-PAGE with an anti-Nup62 antibody showed a single, markedly up-shifted band near the top of the separating gel (Fig 4A, lower panel, and S4 Fig). Treatment of cells with the OGA inhibitor TMG did not further decrease the mobility of Nup62 (S4 Fig). Knockdown of OGT by siRNA dramatically increased the mobility of Nup62 on WGA-SDS-PAGE, resulting in the appearance of a lower band. These findings indicated that, in contrast to AGFG1, almost all Nup62 proteins in cells exist as a homogeneous, highly O-GlcNAc-modified form. Therefore, these combined data indicated that WGA-SDS-PAGE is a useful tool for analysis of the O-GlcNAcylation status of endogenous proteins. It should be noted that, unlike our observation, a previous report has suggested that several different O-GlcNAcylated forms of Nup62 are present in rat brain, as O-GlcNAcylated Nup62 conjugated with PEG-tag was detected as multiple bands in SDS-PAGE [7]. Although the precise reason is unclear, this apparent difference might be caused by cell type-dependent regulation of O-GlcNAcylation or by difference in the detection methods.

It has been reported that, in some cases, certain PTMs such as phosphorylation cause the retardation of the electrophoretic-mobility of target proteins, allowing the detection of modified proteins as up-shifted bands in SDS-PAGE [31–33]. On standard SDS-PAGE, the migration-rate depends on the physicochemical properties of proteins, such as hydrophobicity and net-charge [34]. However, in many cases, O-GlcNAcylation does not induce a significant change in the electrophoretic mobility of the modified protein in standard SDS-PAGE due to the low molecular weight and lack of charge of an O-GlcNAc residue. Hence few techniques for the quantification of O-GlcNAcylated proteins have been developed. In the present study, we demonstrated for the first time that O-GlcNAcylated proteins could be separated from their unmodified forms by electrophoresis through an SDS-polyacrylamide gel containing an immobilized WGA layer, therefore enabling the sensitive and quantitative detection of various O-GlcNAcylated proteins. Previously developed methods for the detection of O-GlcNAcylated proteins require complicated processes, such as *in vitro* enzymatic and chemical reactions, and mass-spectrometry [6, 7]. For example, Rexach *et al*. have recently reported the PEG-tag-mediated detection of O-GlcNAcylated proteins, which requires several specific reagents (e.g., a galactosyltransferase GalT-Y289L mutant, UDP-ketogalactose, and aminooxyl-functiobalized PEG derivatives), as well as time-consuming processes such as GalT-mediated PEGylation and protein purification [7]. In contrast, our WGA-SDS-PAGE method can readily detect and precisely quantifies the O-GlcNAcylation levels of specific proteins without using the unusual chemical materials, and without taking complicated and time-consuming steps. Moreover, this method can be applied to any cellular protein because O-GlcNAcylated protein forms can be detected by immunoblotting using specific antibodies, thereby allowing easy assessment of the O-GlcNAcylaion state of a protein of interest. It should, however, be noted that because WGA binds not only N-acetylglucosamine but also sialic acid, O-GlcNAcylation of slower-migrating proteins in WGA-SDS-PAGE should be confirmed at least once by immunoblotting using the anti-O-GlcNAc antibody or sWGA-HRP as we demonstrated in Fig 2A and S2 Fig. In conclusion, the WGA-SDS-PAGE method can accelerate studies of protein O-GlcNAcylation, and provide novel insights into functions of O-GlcNAcylation in physiological and pathological processes.

## Supporting information

S1 Fig Washing of the gel with transfer buffer containing N-acetylglucosamine did not affect the transfer efficiency of O-GlcNAcylated proteins. HA-Tab1 was transiently expressed with Flag-OGT or Myc-OGA in HEK293 cells, and the cell lysates were separated on WGA-SDS-PAGE (9-mm-long WGA-gel layer containing 3.75 mg/ml of WGA). After electrophoresis was completed, the separating gel was washed three times with transfer buffer with or without 0.5 M N-acetylglucosamine (GlcNAc). Immunoblotting was performed with an anti-HA Ab.
(TIF)

S2 Fig The O-GlcNAcylation of up-shifted bands in WGA-SDS-PAGE is detected by probing with succinylated WGA-HRP. (A) Myc-TFG, (B) Myc-TSC22D1 or (C) Myc-UBAP2L was transiently expressed with Flag-OGT or Flag-OGA in HEK293 cells, and the cell lysates were immunoprecipitated with an anti-Myc Ab. The immune-precipitated proteins or cell lysates were separated on WGA-SDS-PAGE (upper panel, 9-mm-long WGA-gel layer containing 3.75 mg/ml of WGA) or standard SDS-PAGE (lower panel). After electrophoresis and transfer to nitrocellulose membranes were completed, succinylated WGA-HRP (sWGA) or the indicated antibodies were used to probe the membranes.
(TIF)

S3 Fig SiRNA-dependent knockdown of OGT inhibits O-GlcNAcylation of cellular proteins. HEK293 cells were transfected with siRNA targeting OGT or control siRNA, and treated with or without 10 μM Thiamet-G (TMG) for 24 hours. The cell lysates were separated on standard SDS-PAGE, followed by immunoblotting with the indicated antibodies. O-GlcNAcylation levels of cellular proteins were probed with the O-GlcNAc-specific monoclonal antibody, RL2. Actin was used as a loading control.
(TIF)

S4 Fig The effect of different concentrations of WGA-gel on the efficiency of separation of endogenous O-GlcNAc proteins. The effect of WGA concentration on the efficacy of separation of O-GlcNAcylated AGFG1 (top panels) and Nup62 (middle panels). HEK293 cells were transfected with siRNA targeting OGT or control siRNA, and treated with or without 10 μM TMG for 24 hours. The aliquots of HEK293 cell lysates were separated on WGA-SDS-PAGE with different WGA concentrations in the WGA-gel layer. Immunoblotting was performed with the indicated antibodies. Actin was used as a loading control (bottom panel).
(TIF)

## References

[1] Love DC, Hanover JA. The hexosamine signaling pathway: deciphering the "O-GlcNAc code". Science's STKE: signal transduction knowledge environment. 2005;2005(312):re13 doi: 10.1126/stke.3122005re13 . 16317114 10.1126/stke.3122005re13

[2] Hanover JA, Krause MW, Love DC. Bittersweet memories: linking metabolism to epigenetics through O-GlcNAcylation. Nature reviews Molecular cell biology. 2012;13(5):312–21. doi: 10.1038/nrm3334 . 22522719 10.1038/nrm3334

[3] Ruan HB, Singh JP, Li MD, Wu J, Yang X. Cracking the O-GlcNAc code in metabolism. Trends in endocrinology and metabolism: TEM. 2013;24(6):301–9. doi: 10.1016/j.tem.2013.02.002 ; PubMed Central PMCID: PMC3783028. 23647930 10.1016/j.tem.2013.02.002 PMC3783028

[4] Hu P, Shimoji S, Hart GW. Site-specific interplay between O-GlcNAcylation and phosphorylation in cellular regulation. FEBS letters. 2010;584(12):2526–38. doi: 10.1016/j.febslet.2010.04.044 . 20417205 10.1016/j.febslet.2010.04.044

[5] Ma J, Hart GW. O-GlcNAc profiling: from proteins to proteomes. Clinical proteomics. 2014;11(1):8 doi: 10.1186/1559-0275-11-8 ; PubMed Central PMCID: PMC4015695. 24593906 10.1186/1559-0275-11-8 PMC4015695

[6] Shen DL, Gloster TM, Yuzwa SA, Vocadlo DJ. Insights into O-linked N-acetylglucosamine ([0–9]O-GlcNAc) processing and dynamics through kinetic analysis of O-GlcNAc transferase and O-GlcNAcase activity on protein substrates. The Journal of biological chemistry. 2012;287(19):15395–408. doi: 10.1074/jbc.M111.310664 ; PubMed Central PMCID: PMC3346082. 22311971 10.1074/jbc.M111.310664 PMC3346082

[7] Rexach JE, Rogers CJ, Yu SH, Tao J, Sun YE, Hsieh-Wilson LC. Quantification of O-glycosylation stoichiometry and dynamics using resolvable mass tags. Nature chemical biology. 2010;6(9):645–51. doi: 10.1038/nchembio.412 ; PubMed Central PMCID: PMC2924450. 20657584 10.1038/nchembio.412 PMC2924450

[8] Chalkley RJ, Burlingame AL. Identification of GlcNAcylation sites of peptides and α-crystallin using Q-TOF mass spectrometry. Journal of the American Society for Mass Spectrometry. 2001;12(10):1106–13. . 11605972 10.1016/s1044-0305(01)00295-1

[9] Popov M, Li J, Reithmeier RA. Resolution of glycoproteins by a lectin gel-shift assay. Analytical biochemistry. 2000;279(1):90–5. doi: 10.1006/abio.1999.4443 . 10683235 10.1006/abio.1999.4443

[10] Aub JC, Sanford BH, Wang LH. Reactions of normal and leukemic cell surfaces to a wheat germ agglutinin. Proceedings of the National Academy of Sciences of the United States of America. 1965;54(2):400–2. ; PubMed Central PMCID: PMC219677. 5217427 10.1073/pnas.54.2.400 PMC219677

[11] Wright CS. Refinement of the crystal structure of wheat germ agglutinin isolectin 2 at 1.8 Å resolution. Journal of molecular biology. 1987;194(3):501–29. . 3625772 10.1016/0022-2836(87)90678-4

[12] Wright CS. Crystal structure of a wheat germ agglutinin/glycophorin-sialoglycopeptide receptor complex. Structural basis for cooperative lectin-cell binding. The Journal of biological chemistry. 1992;267(20):14345–52. . 1321144

[13] Rodríguez-Romero A, A B., Hernández-Arana A. Unusual far-ultraviolet circular dichroism of wheat germ agglutinin and hevein originated from cystine residues. Biochim Biophys Acta. 1989;998:21–4.

[14] Chavelas EA, Beltrán AP, Pérez-Hernández G, García-Hernández E. Spectroscopic characterization of the thermal unfolding of wheat germ agglutinin. J Mex Chem Soc 2004;48(4):279–82.

[15] Kelly WG, Hart GW. Glycosylation of chromosomal proteins: localization of O-linked N-acetylglucosamine in Drosophila chromatin. Cell. 1989;57(2):243–51. . 2495182 10.1016/0092-8674(89)90962-8

[16] Zachara NE, Vosseller K, Hart GW. Detection and analysis of proteins modified by O-linked N-acetylglucosamine. Current protocols in molecular biology. 2011;Chapter 17:Unit 17 6. doi: 10.1002/0471142727.mb1706s95 ; PubMed Central PMCID: PMC3329785. 21732316 10.1002/0471142727.mb1706s95 PMC3329785

[17] Shibuya H, Yamaguchi K, Shirakabe K, Tonegawa A, Gotoh Y, Ueno N, et al TAB1: an activator of the TAK1 MAPKKK in TGF-β signal transduction. Science. 1996;272(5265):1179–82. . 8638164 10.1126/science.272.5265.1179

[18] Pathak S, Borodkin VS, Albarbarawi O, Campbell DG, Ibrahim A, van Aalten DM. O-GlcNAcylation of TAB1 modulates TAK1-mediated cytokine release. The EMBO journal. 2012;31(6):1394–404. doi: 10.1038/emboj.2012.8 ; PubMed Central PMCID: PMC3321193. 22307082 10.1038/emboj.2012.8 PMC3321193

[19] Teo CF, Ingale S, Wolfert MA, Elsayed GA, Not LG, Chatham JC, et al Glycopeptide-specific monoclonal antibodies suggest new roles for O-GlcNAc. Nature chemical biology. 2010;6(5):338–43. doi: 10.1038/nchembio.338 ; PubMed Central PMCID: PMC2857662. 20305658 10.1038/nchembio.338 PMC2857662

[20] Wang Z, Udeshi ND, Slawson C, Compton PD, Sakabe K, Cheung WD, et al Extensive crosstalk between O-GlcNAcylation and phosphorylation regulates cytokinesis. Science signaling. 2010;3(104):ra2 doi: 10.1126/scisignal.2000526 ; PubMed Central PMCID: PMC2866299. 20068230 10.1126/scisignal.2000526 PMC2866299

[21] Trinidad JC, Barkan DT, Gulledge BF, Thalhammer A, Sali A, Schoepfer R, et al Global identification and characterization of both O-GlcNAcylation and phosphorylation at the murine synapse. Molecular & cellular proteomics: MCP. 2012;11(8):215–29. doi: 10.1074/mcp.O112.018366 ; PubMed Central PMCID: PMC3412957. 22645316 10.1074/mcp.O112.018366 PMC3412957

[22] Monsigny M, Roche AC, Sene C, Maget-Dana R, Delmotte F. Sugar-lectin interactions: how does wheat-germ agglutinin bind sialoglycoconjugates? Eur J Biochem. 1980;104(1):147–53. . 6892800 10.1111/j.1432-1033.1980.tb04410.x

[23] Bogerd HP, Fridell RA, Madore S, Cullen BR. Identification of a novel cellular cofactor for the Rev/Rex class of retroviral regulatory proteins. Cell. 1995;82(3):485–94. . 7634337 10.1016/0092-8674(95)90437-9

[24] Fritz CC, Zapp ML, Green MR. A human nucleoporin-like protein that specifically interacts with HIV Rev. Nature. 1995;376(6540):530–3. doi: 10.1038/376530a0 . 7637788 10.1038/376530a0

[25] Sanchez-Velar N, Udofia EB, Yu Z, Zapp ML. hRIP, a cellular cofactor for Rev function, promotes release of HIV RNAs from the perinuclear region. Genes & development. 2004;18(1):23–34. doi: 10.1101/gad.1149704 ; PubMed Central PMCID: PMC314270. 14701878 10.1101/gad.1149704 PMC314270

[26] Khidekel N, Ficarro SB, Peters EC, Hsieh-Wilson LC. Exploring the O-GlcNAc proteome: direct identification of O-GlcNAc-modified proteins from the brain. Proceedings of the National Academy of Sciences of the United States of America. 2004;101(36):13132–7. doi: 10.1073/pnas.0403471101 ; PubMed Central PMCID: PMC516536. 15340146 10.1073/pnas.0403471101 PMC516536

[27] Myers SA, Panning B, Burlingame AL. Polycomb repressive complex 2 is necessary for the normal site-specific O-GlcNAc distribution in mouse embryonic stem cells. Proceedings of the National Academy of Sciences of the United States of America. 2011;108(23):9490–5. doi: 10.1073/pnas.1019289108 ; PubMed Central PMCID: PMC3111310. 21606357 10.1073/pnas.1019289108 PMC3111310

[28] Ben-Efraim I, Gerace L. Gradient of increasing affinity of importin beta for nucleoporins along the pathway of nuclear import. The Journal of cell biology. 2001;152(2):411–7. ; PubMed Central PMCID: PMC2199621. 11266456 10.1083/jcb.152.2.411 PMC2199621

[29] Lubas WA, Smith M, Starr CM, Hanover JA. Analysis of nuclear pore protein p62 glycosylation. Biochemistry. 1995;34(5):1686–94. . 7849028 10.1021/bi00005a025

[30] Mizuguchi-Hata C, Ogawa Y, Oka M, Yoneda Y. Quantitative regulation of nuclear pore complex proteins by O-GlcNAcylation. Biochimica et biophysica acta. 2013;1833(12):2682–9. doi: 10.1016/j.bbamcr.2013.06.008 . 23777819 10.1016/j.bbamcr.2013.06.008

[31] Barsomian GD, Johnson TL, Borowski M, Denman J, Ollington JF, Hirani S, et al Cloning and expression of peptide-N^4^-(N-acetyl-β-D-glucosaminyl)asparagine amidase F in Escherichia coli. The Journal of biological chemistry. 1990;265(12):6967–72. . 2182635

[32] Kinoshita E, Kinoshita-Kikuta E, Koike T. Separation and detection of large phosphoproteins using Phos-tag SDS-PAGE. Nature protocols. 2009;4(10):1513–21. doi: 10.1038/nprot.2009.154 . 19798084 10.1038/nprot.2009.154

[33] Sarge KD, Park-Sarge OK. Detection of proteins sumoylated in vivo and in vitro. Methods in molecular biology. 2009;590:265–77. doi: 10.1007/978-1-60327-378-7_17 ; PubMed Central PMCID: PMC2755565. 19763510 10.1007/978-1-60327-378-7_17 PMC2755565

[34] Adamson NJ, Reynolds EC. Rules relating electrophoretic mobility, charge and molecular size of peptides and proteins. Journal of chromatography B, Biomedical sciences and applications. 1997;699(1–2):133–47. . 9392373 10.1016/s0378-4347(97)00202-8

