# Supplementary figures and images for: WGA-based lectin affinity gel electrophoresis: A novel method for the detection of O-GlcNAc-modified proteins
Source: PLoS One. 2017 Jul 7;12(7):e0180714. doi: 10.1371/journal.pone.0180714 (PMC5501588; doi:10.1371/journal.pone.0180714)

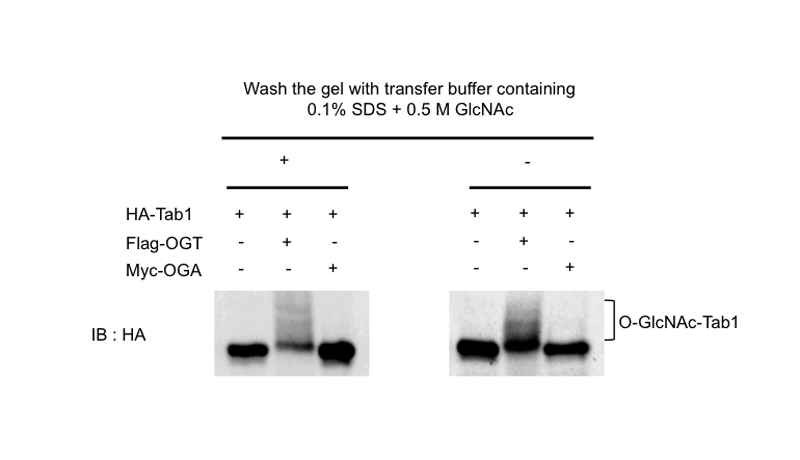

Supplement: S1 Fig — HA-Tab1 was transiently expressed with Flag-OGT or Myc-OGA in HEK293 cells, and the cell lysates were separated on WGA-SDS-PAGE (9-mm-long WGA-gel layer containing 3.75 mg/ml of WGA). After electrophoresis was completed, the separating gel was washed three times with transfer buffer with or without 0.5 M N-acetylglucosamine (GlcNAc). Immunoblotting was performed with an anti-HA Ab. (TIF) [file pone.0180714.s001.tif]

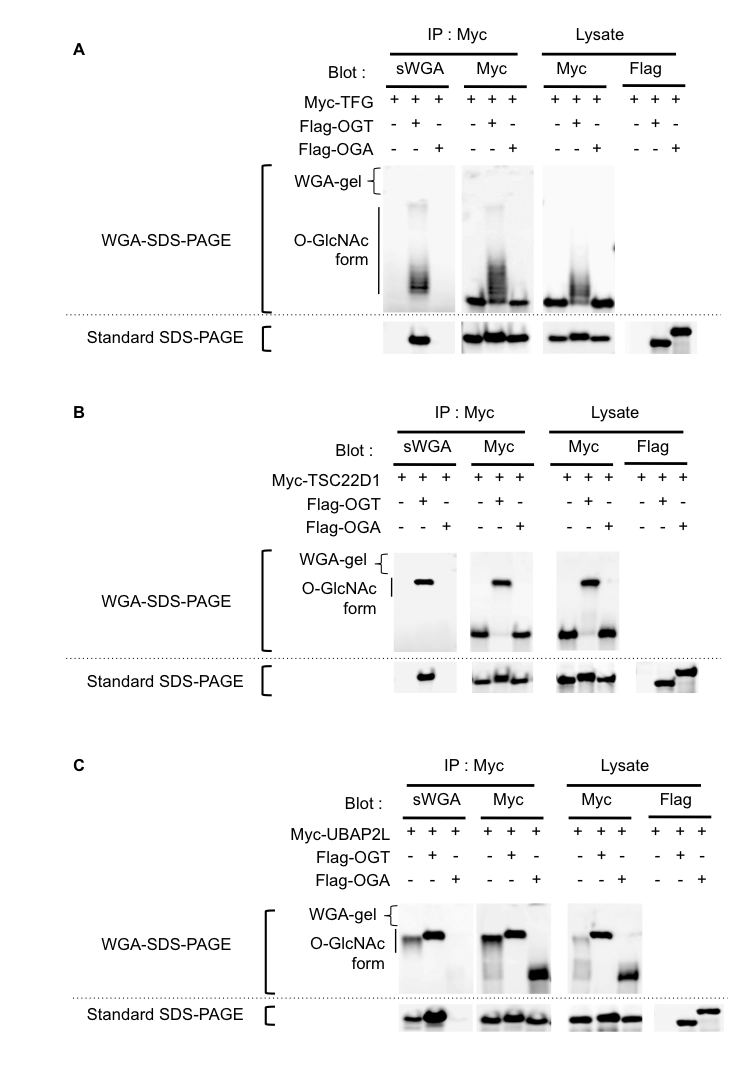

Supplement: S2 Fig — (A) Myc-TFG, (B) Myc-TSC22D1 or (C) Myc-UBAP2L was transiently expressed with Flag-OGT or Flag-OGA in HEK293 cells, and the cell lysates were immunoprecipitated with an anti-Myc Ab. The immune-precipitated proteins or cell lysates were separated on WGA-SDS-PAGE (upper panel, 9-mm-long WGA-gel layer containing 3.75 mg/ml of WGA) or standard SDS-PAGE (lower panel). After electrophoresis and transfer to nitrocellulose membranes were completed, succinylated WGA-HRP (sWGA) or the indicated antibodies were used to probe the membranes. (TIF) [file pone.0180714.s002.tif]

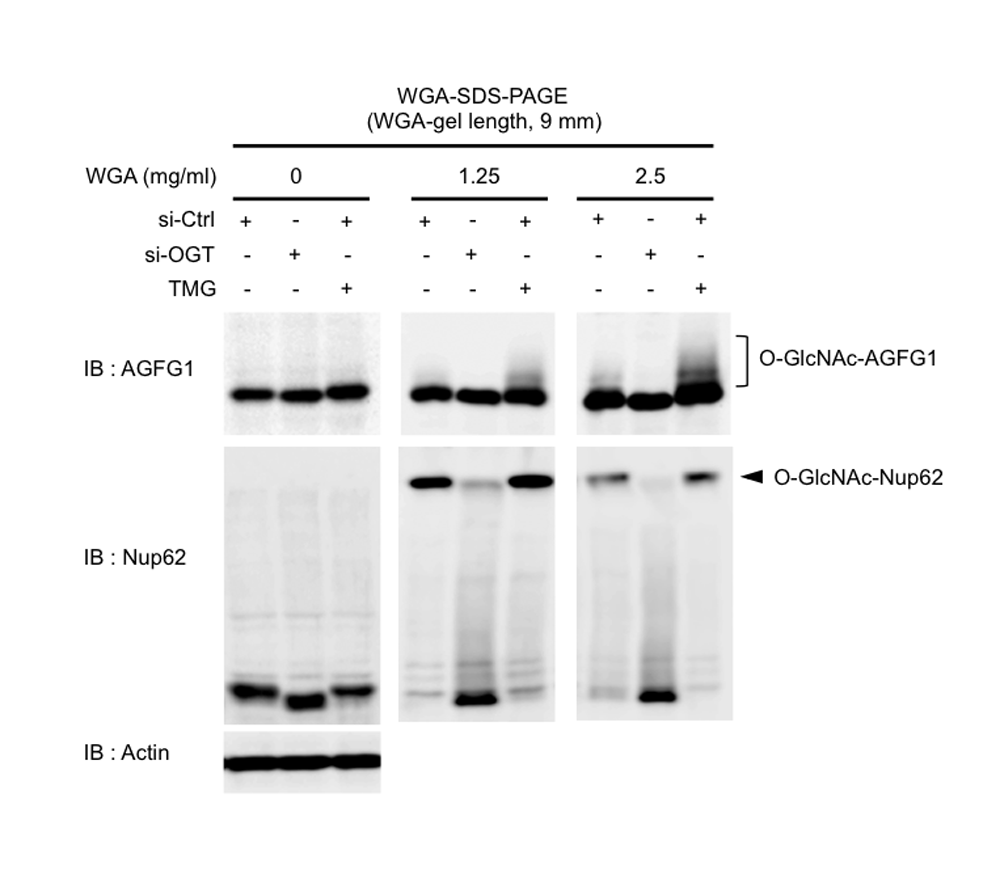

Supplement: S4 Fig — The effect of WGA concentration on the efficacy of separation of O-GlcNAcylated AGFG1 (top panels) and Nup62 (middle panels). HEK293 cells were transfected with siRNA targeting OGT or control siRNA, and treated with or without 10 μM TMG for 24 hours. The aliquots of HEK293 cell lysates were separated on WGA-SDS-PAGE with different WGA concentrations in the WGA-gel layer. Immunoblotting was performed with the indicated antibodies. Actin was used as a loading control (bottom panel). (TIF) [file pone.0180714.s004.tif]
